# Including educational status may improve cardiovascular risk calculations such as SCORE2

**DOI:** 10.3389/fcvm.2024.1345277

**Published:** 2024-10-11

**Authors:** Christiane Dienhart, Isabella Gostner, Vanessa Frey, Elmar Aigner, Bernhard Iglseder, Patrick Langthaler, Bernhard Paulweber, Eugen Trinka, Bernhard Wernly

**Affiliations:** ^1^ Department of Internal Medicine I, Paracelsus Medical University, Salzburg, Austria; ^2^ Department of Cardiology & Nephrology, Salzkammergut Klinikum Vöcklabruck, Vöcklabruck, Austria; ^3^ Department of Neurology, Christian Doppler University Hospital, Paracelsus Medical University and Centre for Cognitive Neuroscience, Affiliated Member of the European Reference Network EpiCARE, Salzburg, Austria; ^4^ Department of Geriatric Medicine, Christian Doppler University Hospital, Paracelsus Medical University, Salzburg, Austria; ^5^ Department of Artificial Intelligence and Human Interfaces, Paris Lodron University of Salzburg, Salzburg, Austria; ^6^ Team Biostatistics and Big Medical Data, IDA Lab Salzburg, Paracelsus Medical University Salzburg, Salzburg, Austria; ^7^ Obesity Research Unit, Paracelsus Medical University, Salzburg, Austria; ^8^ Neuroscience Institute, Christian Doppler University Hospital, Paracelsus Medical University and Centre for Cognitive Neuroscience, Salzburg, Austria; ^9^ Department of Public Health, Health Services Research and Health Technology Assessment, UMIT—University for Health Sciences, Medical Informatics and Technology, Hall in Tirol, Austria; ^10^ Department of Internal Medicine I, Oberndorf Hospital, Salzburg, Austria; ^11^ Institute for General and Preventive Medicine, Paracelsus Medical University, Salzburg, Austria

**Keywords:** morbidity and mortality, cardiovascular health, carotid doppler duplex, plaque, educational status, SCORE2

## Abstract

**Background:**

The association between education and atherosclerotic cardiovascular disease (ASCVD) has been well described for decades. Nevertheless, most cardiovascular risk models, including SCORE2, still do not take educational status into account even if this factor is easily assessed and costs nothing to acquire. Using carotid plaques as a proxy for ASCVD, we analysed educational status as associated with carotid plaque development, to determine if the relationship remains, how it relates to traditional risk factors and, how it impacts the European cardiovascular risk model, SCORE2. Our study also provides further data on plaque development in a well-characterised population nearly equally weighted by gender.

**Methods:**

9,083 subjects (51% female, 49% male) from the Paracelsus 10,000 cohort, underwent a carotid doppler duplex as part of thorough screening for subclinical ASCVD. Well over 90% of carotid doppler duplex examinations were performed by the same experienced clinician. Subjects were then classified by educational status using the Generalized International Standard Classification of Education. Plaque absence or presence was dichotomised and variables analysed using regression modelling to examine educational status relative to cardiovascular risk factors and with respect to the SCORE2 model.

**Results:**

Using medium educational status as a reference, subjects in our cohort with low educational status had higher odds, while subjects with high educational status had lower odds for carotid plaques compared to subjects with medium education (aOR 1.76 95%CI 1.50–2.06; and 0.0.63 95%CI 0.57–0.70, respectively). Even after adjusting for common risk factors including metabolic syndrome and SCORE2, the relationship was maintained. Furthermore, when comparing the potential predictive power of SCORE2 alone and plus educational status using the Akaike information criterion, we showed a ‘better fit’ when educational status was added.

**Conclusions:**

Measuring educational status is cost-free and easy for clinicians to obtain. We believe cardiovascular risk prediction models such as SCORE2 may more accurately reflect individual risk if educational status is also taken into account. Additionally, we believe clinicians need to understand and appropriately address educational status as a risk factor, to better quantify individual risk and take appropriate measures to reduce risk so that the association may finally be broken.

## 1 Introduction

Although improvements in mortality and incidence have been made, atherosclerotic cardiovascular heart disease (ASCVD) is a leading cause of morbidity and mortality worldwide, causing over one third of deaths in the EU and costing over €200 billion per year (1). Furthermore, according to a joint publication from the European Heart Network (EHN) and the European Society of Cardiology (ESC), approx. 20%–40% of heart attacks occur in patients who were unaware of their CVD diagnosis (2). Thus, it is important to identify individuals and groups with elevated cardiovascular risk as early as possible to initiate targeted risk reduction measures. Furthermore, it is imperative to better understand personal risk in order to address the patient as an individual. Educational status is a cost free and easily gatherable data point which should not be ignored by clinicians in practice.

Socioeconomic status is one of several significant risk factors, which contributes to an individual's risk of developing ASCVD. As educational status tends to reflect an individual's access to resources, financial stability, and social standing, this might be a separate, and, maybe, better marker for assessing health risk, than, for example, income (3, 4). Numerous studies have identified an association between lower educational status and poor health outcomes, including an increased risk of ASCVD, with some of these studies having been published decades ago (5–8). However, the relationship persists (9–14). We have already analysed a portion of our Paracelsus 10,000 cohort based on CT calcium scores (CACS) and found that education and coronary calcium seem to be linked inversely (15). As it has been shown that ignoring educational disparities may bias health expectancy measures (16), we aim to further explore this relationship with respect to cardiovascular risk scores, particularly SCORE2 (17).

Numerous studies have shown that CDD (doppler duplex ultrasound of the carotid arteries) plaques are predictors of significant ASCVD (18–20). The European Society of Cardiology (ESC) recommends the use of either CACS or CDD in addition to a standard cardiovascular risk calculator to help quantify cardiovascular risk especially in asymptomatic intermediate risk patients. While CACS is preferable, CDD can be used in the cases where resources and access to CT is limited (21).

Thus, we aim to investigate the relationship between CDD plaques and common traditional risk factors, with a focus on educational status in our middle European population. By exploring this association, especially with respect to the ESC recommended SCORE2 risk calculation, we hope to bring this issue to the forefront once again, as well as provide additional valuable insights that could inform public health strategies and policies and thus have significant implications for both population-level interventions and individual patient care. By better understanding the relationship between educational status and CDD plaques, healthcare providers can make better informed decisions when recommending screening and preventive measures for their patients, especially in the context of ESC recommendations.

## 2 Subjects, materials and methods

### 2.1 Subjects

The Paracelsus 10,000 study is a prospective, regional Salzburg-based study, in which a cohort of men and women, aged 40–77 years, were recruited randomly from a local population registry. Of the 56,595 invited participants, 9,758 attended initial visits within a 2013–2020 timeframe. A further 286 participants were not invited but requested to partake in the study; these included the 15 participants who were aged <40 years old. All study participants were subjected to a screening program that included collection of a detailed personal and family history, a physical examination, as well as various anthropometric clinical and laboratory measures (15, 22). For a detailed explanation of the Paracelsus 10,000 study and its methods, we refer to the paper by Frey et al. (22) Follow-up has recently started and will be ongoing.

Most study participants also underwent a CDD, previously described in Dienhart et al. (23) as follows: Well over 90% of the ultrasound examinations were performed by the same experienced operator. Ultrasounds of both carotid arteries were performed in a supine position using the same Panasonic GM−72P00A machine (Panasonic Healthcare Diagnostics US) for all examinations. Plaques were defined as deposits on the vessel wall with a diameter of >1.5 mm, as well as an area >2.9 mm^2^. Multiple measurements of each plaque were performed from various transducer positions to increase accuracy. Plaque morphology was defined using the Gray-Weale score (types 1–4) (24) Stenosis was recorded if there was a reduction in the vessel lumen of >20%–30% according to ECST guidelines. Total plaque area was defined as the summation of all plaque surfaces of the common carotid artery, the internal carotid artery (bulb and proximal course), as well as the external carotid artery of the respective side (plaque area left, plaque area right). All images were stored on the hospital imaging system for future reference. Results were also recorded in the Paracelsus 10,000 data bank.

For our analysis, we included only subjects who had complete data available on educational status, a full ultrasound analysis and all the data necessary to complete a SCORE2 calculation. These 9,083 subjects were grouped into three classes of educational status (high, medium and low) using the Generalised International Standard Classification of Education (GISCED) based on methods described in Figure 1 of Schneider et al. (23) Subjects who had post-high school studies including vocational school equivalent to a bachelors degree or higher were labelled as “high” educational status. Subjects with either vocational education and training equivalent to upper secondary school/high school were labelled as “medium” educational status. Subjects, who had at most completed compulsory education, were classified as “low” education status (15).

**Figure 1:**
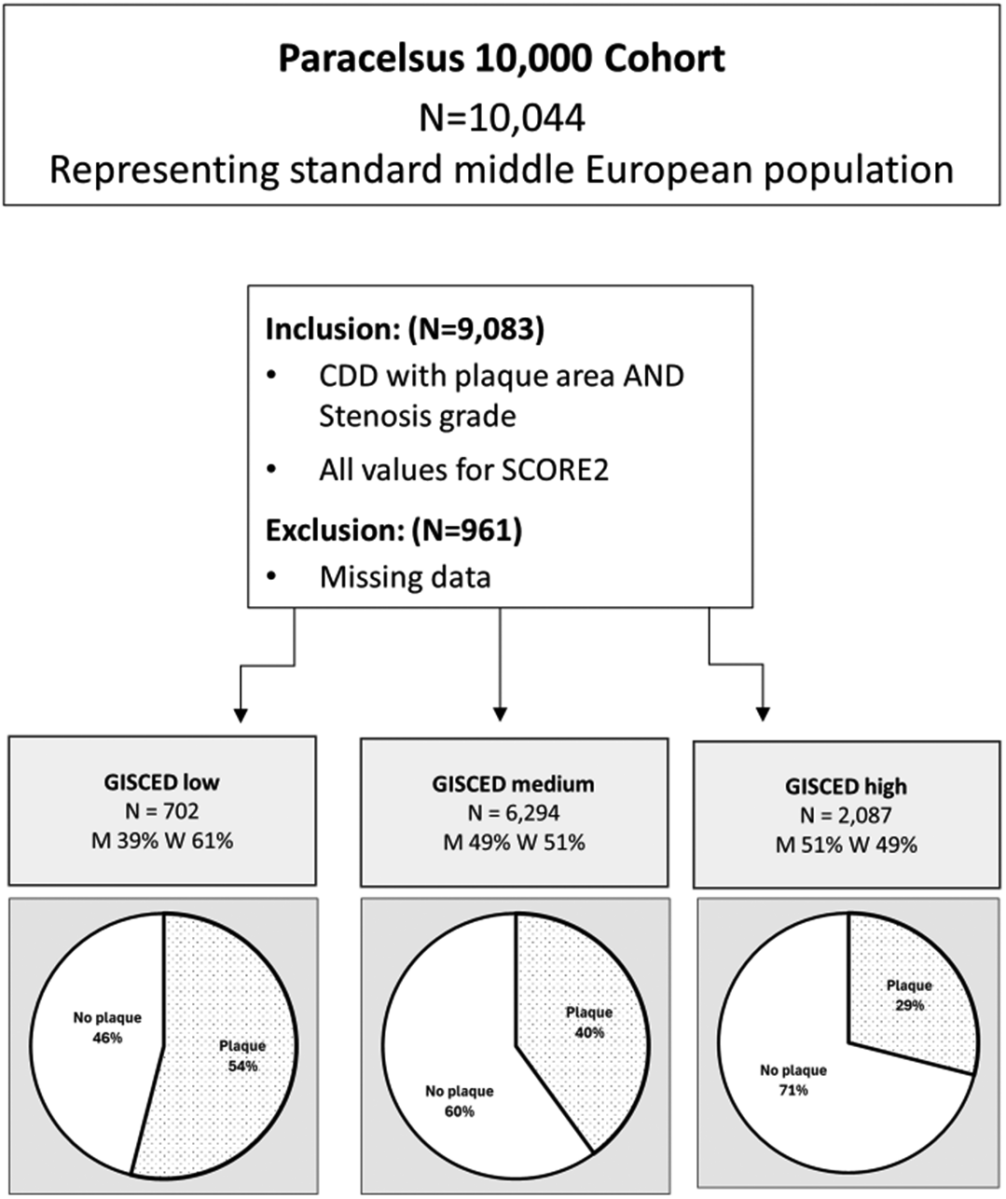
GISCED stratification of participants with inclusion criteria.

### 2.2 Statistical analysis

Subjects were stratified by educational status (high, medium, low) to evaluate differences in baseline characteristics. Data are presented as number (*N*) and percent (%) for categorical variables and as median with interquartile range (IQR) for continuous variables to take into account that the data were not normally distributed. In order to analyse the effects of the educational status on carotid plaques, plaques were categorised dichotomously as either 0 or 1 with presence of plaques as the dependent variable in the logistic regression models. The relationship between carotid plaques and educational status was analysed using both univariate and multivariable analysis. For the univariate analysis, we have used Kruksal–Wallis to analyse continuous variables and Chi Square for categorical variables. We also performed a logistic regression for multivariable analysis. We fitted multiple models: beyond the baseline model, Model-1 adjusted for age and sex, model-2 for age, sex and the concomitant diagnosis of metabolic syndrome, and model-3 SCORE2, the ESC's cardiovascular risk assessment tool (17). Model-4 was adjusted for age, sex, body mass index (BMI), low density lipid (LDL) cholesterol level and self-reported hypertension and or diabetes mellitus type 2 in the medical history. A final model (model-5) adjusted for the factors in model-4 as well as for household income according to the stratification applied in the Paracelsus 10,000 survey. We calculated adjusted odds ratios (aORs) and the respective 95% confidence intervals (CI). A *p*-value of <0.05 was deemed significant. All tests were performed as two-sided.

We also performed a receiver operator characteristic (ROC) Analysis to calculate area under the curve (AUC) using SCORE2 and SCORE2 plus educational status to predict the likelihood of plaques in our population. To compare the models and determine best fit, we used an Akaike information criterion analysis (25).

Statistics were calculated using Stata (StataCorp, USA). SCORE2 was calculated in Stata using the script provided by the authors of the SCORE2 working group as detailed in the paper by Hagemann et al. (17).

## 3 Results

According to self-reported gender, our study population (*N* = 9,083) was nearly equally distributed between men (49%) and women (51%), with an overall median age of 55 years. The vast majority (92%) of our cohort had acquired at least “medium” educational status (equivalent to a minimum of upper secondary school/high school or more). In the lowest educational status, women were overrepresented (61%), while at the highest level of education, men held a slight majority (51%). A further descriptive demographic overview is included in Table 1.

**Table 1:** Descriptive overview of 9,083 subjects with CDD in paracelsus 10,000 cohort.

	GISCED = low	GISCED = medium	GISCED = high	*p*-value
	*N* = 702	*N* = 6,294	*N* = 2,087	
Age	59 (52–65)	55 (50–61)	54 (47–60)	<0.001
Age <40	0% (2)	0% (6)	0% (7)	
Age 40–49	17% (116)	23% (1,478)	31% (653)	
Age 50–59	34% (239)	44% (2,787)	42% (877)	
Age 60–69	39% (276)	28% (1,781)	24% (497)	
Age ≥70	10% (69)	4% (242)	3% (53)	
By self-reported gender				<0.001
Men	39% (273)	49% (3,077)	51% (1,071)	
Women	61% (429)	51% (3,217)	49% (1,016)	
HbA1c (%)	5.5 (5.4–5.8)	5.4 (5.3–5.6)	5.4 (5.2–5.6)	<0.001
Total choleserol (mg/dl)	214 (186–237)	209 (184–234)	208 (184–234)	0.096
Triglycerides (mg/dl)	110 (81–147)	97 (71–137)	91 (68–128)	<0.001
HDL cholesterol (mg/dl)	58 (48–71)	62 (50–75)	62 (52–75)	<0.001
LDL cholesterol (mg/dl)	144 (117–168)	139 (116–165)	139 (116–163)	0.057
Height (cm)	167 (160–174)	171 (164–177)	173 (166–179)	<0.001
Weight (kg)	76 (66–87)	77 (66–88)	75 (65–85)	<0.001
Body mass index (BMI)	27 (24–30)	26 (23–29)	25 (23–27)	<0.001
BMI <18.5	1% (9)	1% (56)	1% (26)	
BMI 19.5–24.9	28% (199)	40% (2,488)	49% (1,020)	
BMI 25–29.9	40% (283)	39% (2,479)	38% (785)	
BMI 30–34.9	22% (151)	15% (941)	10% (211)	
BMI 35–39.9	6% (42)	4% (241)	2% (35)	
BMI ≥40	3% (18)	1% (89)	0% (9)	
Abdominal circumf. (cm)	95 (87–105)	93 (84–102)	90 (82–98)	<0.001
Central obesity	53% (371)	40% (2,541)	29% (615)	<0.001
Metabolic syndrome^a^	25% (172)	17% (1,096)	11% (238)	<0.001
Self-reported				
Dyslipidemia	18% (125)	12% (756)	10% (198)	<0.001
Diabetes mellitus type 2	7% (46)	3% (216)	2% (47)	<0.001
Hypertension	35% (242)	23% (1,425)	16% (329)	<0.001
Coronary artery disease	4% (28)	2% (115)	1% (31)	<0.001
Chronic heart failure	1% (7)	0% (30)	1% (11)	0.20
Peripheral vascular disease	1% (7)	0% (27)	0% (1)	0.001
COPD	3% (18)	2% (117)	1% (23)	0.016
Chronic kidney disease	0% (3)	0% (23)	0% (9)	0.90
Never smoker	38% (270)	43% (2,680)	54% (1,131)	<0.001
Previous smoking	35% (248)	38% (2,416)	32% (676)	<0.001
Current smoker	26% (184)	19% (1,198)	13% (280)	<0.001
SCORE2 10 years CVD risk (%)	5.4 (3.1–8.5)	4.1 (2.2–6.9)	3.3 (1.8–5.8)	<0.001

^a^ Metabolic syndrome according to International Diabetes Federation Criteria.

As expected, the presence of carotid plaques rises with age and is more common in males. Furthermore, participants with higher levels of educational status were statistically significantly younger than those with higher educational status. Thus, we adjusted all models for age and sex. Nevertheless, after adjusting for age and sex, a higher level of education was still associated with lower odds of carotid plaques (aOR 0.67; 95%CI 0.60–0.76, *p* < 0.001 than in subjects with a low educational status (aOR 0.1.49; 95%CI 1.24–1.79 *p* < 0.001) for subjects high and low educational status, respectively, vs. medium educational status. Not only was presence of plaques associated inversely with educational status, but total plaque area also increased as educational status decreased (see Table 2 for further reference).

**Table 2:** Analysis of population by stenosis grade and plaque area.

Any plaque	GISCED = low	GISCED = med	GISCED = high	<0.001
None	46% (330)	60% (3,803)	71% (1,475)	
Present	54% (376)	40% (2,491)	29% (612)	
Stenosis gradient				<0.001
No stenosis	58% (407)	74% (4,660)	81% (1,678)	
ECST <50%	41% (290)	25% (1,594)	19% (397)	
ECST 50%–69%	1% (4)	0% (23)	0% (7)	<0.001
ECST 70%–80%	0% (0)	0% (5)	0% (1)	
ECST >80%	0% (0)	0% (5)	0% (0)	
Total plaque area	5.09 (0.00–21.85)	0.00 (0.00–11.42)	0.00 (0.00–5.02)	<0.001

Our analysis showed a statistically significant association between educational status and many traditional risk factors associated with ASCVD in the univariate analysis, including HbA1c, total cholesterol, triglycerides and low high-density lipids (HDL), although median HbA1c, triglycerides and HDL were all within the normal ranges in all levels of educational status. Median low density lipid (LDL) cholesterol levels were marginally statistically significantly different between the groups, and, at 144 mg/dl (IQR: 116–165 mg/dl), 139 mg/dl (IQR: 116–165 mg/dl) and 139 mg/dl (IQR: 116–163 mg/dl); *p* = 0.057 (low medium, and high educational status, respectively) were above the upper limit of normal, as well as above the ESC recommended limits (21). SCORE2 also decreased as educational status increased (5.4 (IQR: 3.1–8.5), 4.1 (IQR: 2.2–6.9) and 3.3 (IQR: 1.8–5.8); *p* = 0.001).

Results from our study show a statistically significant inverse relationship between educational status, weight, height, BMI and abdominal circumference, as well as central obesity. Furthermore, self-reported diagnoses of diabetes mellitus, hypertension, coronary artery disease, peripheral artery disease (PAD) and chronic obstructive pulmonary disease (COPD) statistically increased as educational levels decreased.

The number of self-reported current smokers (26%, 19%, 13%; *p* ≤ 0.001) as well as the number of subjects having ever smoked cigarettes (48%, 47%, 37%; *p* ≤ 0.001), was statistically significantly higher in the group with low educational status compared to higher educational status. Furthermore, subjects with low educational status were also more likely to have previously smoked cigarettes or to report to currently smoking. (61%, 57%, 46%; *p* ≤ 0.001).

In our multivariable regression models, using medium educational status as a reference, subjects with low education status had higher odds for plaques while those with high educational status had lower odds for carotid plaques compared to subjects with medium education at baseline (aOR 1.76 95%CI 1.51–2.06 vs. aOR 0.63 95% CI 0.57–0.70; *p* < 0.001). All 5 of our models, which made adjustments for age, sex, and various classically known risk factors including metabolic syndrome and SCORE2 risk showed a clear inverse relationship between educational status and the presence of carotid plaques. This relationship was maintained even after adjustment for income levels, which were stratified according to income levels as per the income questionnaires as collected at the initial visit. (See Table 3: Logistic regression: Odds of any plaque using medium educational status as reference for a further analysis in detail).

**Table 3:** Logistic regression: odds of any plaque using medium educational status as reference.

Education Status	GISCED = medium	GISCED = low	GISCED = high
Baseline	ref	aOR 1.76; 95%CI 1.50–2.06; *p* < 0.001	aOR 0.63; 95%CI 0.57–0.70; *p* < 0.001
Model 1	ref	aOR 1.49; 95%CI 1.24–1.79; *p* < 0.001	aOR 0.67; 95%CI 0.60–0.76; *p* < 0.001
Model 2	ref	aOR 1.44; 95%CI 1.20–1.73; *p* < 0.001	aOR 0.69; 95%CI 0.61–0.78; *p* < 0.001
Model 3	ref	aOR 1.36; 95%CI 1.14–1.63; *p* = 0.001	aOR 0.72; 95%CI 0.64–0.82; *p* < 0.001
Model 4	ref	aOR 1.35; 95%CI 1.12–1.63; *p* = 0.001	aOR 0.71; 95%CI 0.63–0.80; *p* < 0.001
Model 5	ref	aOR 1.30; 95%CI 1.08–1.60; *p* = 0.006	aOR 0.73; 95%CI 0.65–0.84; *p* < 0.001

Model 1: adjustment for age and sex.

Model 2: adjustment for age, sex, and metabolic syndrome.

Model 3: adjustment for SCORE2.

Model 4: adjustment for age, sex, self-reported hypertension and DM2, LDL cholesterol and BMI.

Model 5: adjustment for age, sex, self-reported hypertension and DM2, LDL cholesterol, BMI and Income.

In addition to multivariable regression, we analysed the potential predictive power of SCORE2 alone and with the addition of education using an area under the curve (AUC) analysis. Although the AUC did not show a substantial difference (0.7991 and 0.7998, respectively) and the standard error was identical, an analysis based on Akaike information criterion (25), which assists in choosing the “best” model, showed a ‘better fit’ when educational status was added to the model, falling from 9,717 to 9,675 with the additional of educational status.

## 4 Discussion

The inverse relationship between cardiovascular disease and education has been described in the literature for many decades. However, although educational status is a free and easily generated variable, it is not taken into account in daily clinical practice and not reflected in risk modelling. Perhaps, because we have failed to address this issue appropriately, this association continues to persist. Using carotid artery plaques as a proxy for ASCVD and GISCED to measure educational status, our analysis indicates that the inverse relationship between educational status and ASCVD, continues to be maintained in our middle European population. Conversely to the results of our previous study using coronary calcium scores (15), this larger study did show an inverse association between traditional risk factors including HbA1c, total cholesterol, triglycerides and low high density lipids (HDL) upon univariate analysis. However, the inverse association between educational status and CDD plaques remained significant even after adjustment for SCORE2 as well as metabolic risk factors, which take these into account. Furthermore, even when adjusting for income levels, the inverse relationship between educational status and plaques was maintained. Although our analysis showed no difference in the AUC between SCORE2 and SCORE2 plus education, the Akaike analysis showed a ‘better fit’ of a model including education. Thus, our data suggest that the association between educational status and ASCVD risk is only partly mediated by the impact of education on classical ASCVD risk factors. Thus, we believe that educational status should be considered as an added risk factor in predictive models.

Education may be a better factor to take into account social and health care inequalities than income, particularly in European countries. Based on US analyses, it has been postulated that the inverse relationship between health and education is associated with health and income inequalities particularly related to the high education and insurance costs in the US, as well as lack of access to health care caused by socioeconomic status differences (9). However, numerous European studies have also shown an inverse relationship between educational levels and cardiovascular health, despite a lower rate of health inequality and greater social equality in most of the countries under study (26–30). Furthermore, a US study relates lower education and social status to less healthy cardiovascular lifestyle factors based on the AHA recommended ’simple seven (31).

One explanation for the relationship between lower education status, carotid plaques and increased ASCVD risk might be health literacy, which is described in a Swedish study (32). In 2018, the AHA released a scientific statement as to the importance of health literacy both in primary as well as secondary prevention in ASCVD (33). The AHA states that health literacy is a problem not of the individual but of the organisation and has put initiatives in place to address health literacy in America (33). In comparison, in a Pubmed search performed 20.09.2023 using the key words European Society of Cardiology and health literacy, we found no publications addressing health literacy from the European Society of Cardiology. After the Health Literacy Survey in Europe (2011) showed that over 55% of Austrians had an inadequate or problematic health literacy, government initiatives to improve access to health information, improve health related communication and, more effectively promote health initiatives, were set in practice (34). While not exactly comparable due to changes in the questionnaires, the latest survey (2020), showed improvement: just over 15% of Austrians had an inadequate or problematic health literacy. However, it was found that in persons with low educational status and low socioeconomic status, health literacy remained especially low (35). Unfortunately, we did not collect data on the health literacy of our subjects during the initial visits, but our data indicate that assessing educational status, which is free and easy for all clinicians to collect, could give a similar result.

Several studies have indicated that the relationship between educational status and cardiovascular disease is mediated by differences in BMI, metabolic syndrome, diabetes and, particularly hypertension (29), which are also taken into account in traditional models. Our data confirmed a statistically significant inverse relationship between BMI and abdominal circumference, as well as central obesity, and educational status. Furthermore, in our cohort, self-reported diagnoses of diabetes mellitus, hypertension, coronary artery disease, peripheral artery disease and COPD, which are known factors associated with an increase in ASCVD risk, statistically increased as educational status decreased. However, the association between educational status and plaques remained statistically significant after multivariable adjustment for the concomitant diagnosis of metabolic syndrome, and its components as well as reported diabetes mellitus type 2 in past/current medical history.

Supporting the previously described relationship between educational status and smoking (25), in our cohort, the number of self-reported current smokers as well as the number of subjects having ever smoked cigarettes, was also statistically significantly higher in the group with low educational status compared to higher educational status. Additionally, subjects in the group with the lowest educational status were more likely to remain smokers over time. However, even when these factors were adjusted for as part of SCORE2, educational status remained as an independent risk factor. Even after adjusting for income, an independent relationship between educational status and cardiovascular disease remained.

As far as we are aware, we are one of the first groups to analyse SCORE2, which takes into account variables including age, sex, cholesterol, blood pressure and smoking in groups according to educational status in a large European cohort. Along with some of its neighbors (i.e., Germany, Italy and Slovenia), Austria is classified as moderate risk in the SCORE2 model, based on a cardiovascular mortality of 130.9/100,000 according to 2016 reports (17). According to Timmis et all, the incidence of ischemic cardiovascular disease in Austria is approximately 215/100,000 while the prevalence is 1,788/100,000, providing a ranking of 18th and 14th out of 56 countries based on incidence and prevalence, respectively (36). While OECD data suggests Austria has an intermediate level of health inequality, inequalities in perceived unmet needs and unmet needs due to cost are both low (37). Furthermore, Austria has a comparatively low level of income inequality among OECD countries (38). The low income inequality and reasonable health equality may explain why despite a correction for income, higher educational status is still inversely associated with atherosclerotic disease, but it does not explain how this relationship is maintained.

Our cohort showed a statistically significant difference between groups depending on educational status. However, even after adjusting for SCORE2 in our logistic regression model, we showed a continued difference in carotid plaques, which we believe indicates that educational status may be a further risk factor for ASCVD which is not adequately reflected in common risk models. The relationship between ASCVD and educational status might go beyond the increase in cardiometabolic risk factors among socioeconomically weaker subjects and may be a better way to take these factors into account. Furthermore, this risk variable is free and easy to assess during a clinical visit.

Arguments for the maintenance of the association between higher risk for atherosclerotic disease and lower education despite extensive multivariable adjustment, including classical cardiovascular risk factors and income, might come from differences in physical activity, nutrition and sleep patterns (26, 39, 40). For example, a US study has shown disparities in physical activity based on educational status in a US population (41). Previous studies have also shown a link between higher educational status and positive dietary choices, such as increased fiber intake, combined with reduced intake of starch and refined sugars (42). In Germany, a recent study showed that educational status was the most important factor in the consumption of animal products in the population (43). In addition, studies have shown that socioeconomic status can affect sleep duration and sleep disturbance, which may be related to increased ASCVD risk (39). Levels of depression/mental illness, negative childhood experiences and increased stress may also be associated with educational status (27, 28, 44). The analysis of these is unfortunately beyond the scope of this study but needs to be investigated further.

We have performed a cross sectional analysis of CDD plaques in our population and argue that these can be used as a proxy for ASCVD risk. While we understand that there have been issues in quantifying CDD plaques, we believe that our use of a binomial analysis, as well as the fact that the vast majority of CDD were performed by a single operator, make our data more robust. However, while the relationship between CDD plaques and ASCVD is well established, not all of our subjects are destined to develop ASCVD morbidity and mortality. Nevertheless, we believe the arguments for plaques as a harbinger of ASCVD are well documented in the literature. Unfortunately, a clearer description of the relationship between educational status and ASCVD is beyond the scope of this study. There are many factors that may be affected by educational status that need to be further elucidated. Despite all that is already known, we believe that the association between educational status, ASCVD and risk measures needs to be further analysed particularly in longitudinal studies.

## 5 Conclusion

Not only does our study support the association between ASCVD and educational status that continues to persist across nations and time, but we believe that our results indicate that educational status may improve the SCORE2 risk calculation particularly on an individual level. Using medium educational status as a reference, subjects in our cohort with low educational status had higher odds, while subjects with high educational status had lower odds for carotid plaques compared to subjects with medium education. Furthermore, using an Akaike information criterion analysis, we showed that although the AUC and standard error were not significantly different between SCORE2 and SCORE2 + educational status, the goodness of fit was improved. Particularly, as educational status is a factor which costs nothing to acquire and is easily assessed, we believe it is to the patients’ detriment that we continue to ignore this risk factor. Furthermore, we need to make a concerted effort to address this issue on a public health level so that the association may finally be broken.

## Data Availability

The raw data supporting the conclusions of this article will be made available by the authors, without undue reservation.
